# Envisioning the Future of Fine Dining: Insights from a Multi-Methods Study in Germany

**DOI:** 10.3390/foods14132294

**Published:** 2025-06-28

**Authors:** Yana Subbotina-Dubinski, Claus-Christian Carbon

**Affiliations:** Department for General Psychology and Methodology, University of Bamberg, Markusplatz 3, 96047 Bamberg, Germany; ysubbotina@yahoo.com

**Keywords:** fine dining, seminal restaurant concepts, sustainability, seasonality, regionality, prediction, future concepts, nutrition, experts

## Abstract

This article investigates predicted future developments in fine dining using a mixed-methods approach rooted in German gastronomic culture. By conducting an inductive media content analysis and ten semi-structured expert interviews with leading figures in Germany’s high-end food sector, we applied a qualitative mixed-methods approach. The study was based exclusively on data collected in 2018 and 2019, deliberately excluding pandemic-related developments in order to focus on long-term structural and cultural trends in fine dining. We identified two core thematic clusters: one related to sustainable food practices (ecology/sustainability, regionality, seasonality, from-farm-to-table, and vegetarianism/veganism) and the other to experiential dimensions of dining (experience, topic-based concept, and storytelling). Our findings contribute to the academic discussion on culinary futures and provide grounded insights into how fine dining is likely to evolve in response to broader societal, environmental, and cultural shifts. This study fills a significant research gap by systematically mapping emerging restaurant concepts based on non-COVID data, making it a valuable reference for scholars and practitioners alike.

## 1. Introduction

Fine dining, as a segment of haute cuisine, serves as a barometer for innovation and cultural transformation within the broader hospitality industry [1]. Despite its influence, there remains a surprising scarcity of systematic research into the evolving concepts that may shape its future [2]. Existing research has examined fine dining through various lenses—focusing on innovation processes [3,4], customer motivations and perceptions [5,6,7], and the growing interplay between gastronomy and scientific or societal discourse [8,9]. Haute cuisine has increasingly been understood as a creative and collaborative field that facilitates innovation not only within individual kitchens but also across networks and gastronomic clusters [10]. Pioneers like Ferran Adrià have exemplified how fine dining can drive institutional change through culinary innovation and conceptual disruption [11]. While these contributions provide valuable insights, they tend to isolate specific aspects rather than explore how conceptual restaurant strategies are evolving in response to broader ecological and cultural transformations. Moreover, a gap remains in understanding how such innovations coalesce into future-oriented restaurant models, particularly within specific national contexts like Germany. From an organizational perspective, innovation in fine dining is not only about creativity but also about maintaining legitimacy in a highly codified culinary field. A recent study shows how Michelin-starred chefs navigate this tension through strategic innovation processes [12]. These findings underscore that future-oriented innovation in fine dining depends not only on originality but also on maintaining symbolic legitimacy within the field. Typically, studies deal with specific questions in this area, for instance, how evidence-based scientific knowledge has found its way into modern fine dining [8] or how the decision-making processes for a particular gourmet restaurant from an economic perspective work [13]. Creativity in *haute cuisine* [4] and the influence of acoustic factors in gourmet restaurants [14] have also been studied. Ottenbacher and Harrington [3] noted that, for the innovation development process of Michelin-starred chefs, in contrast to other product innovation processes, the human factor plays an overriding role in fine dining. Some working groups dealt with such questions in a country-specific manner [2,9]. However, certain specific issues related to restaurant concepts in fine dining have already been investigated [15,16,17]. Recent research emphasizes that sustainable practices are increasingly shaping the future of high-end dining—not only as ethical imperatives but also as key elements of memorable customer experiences [18]. In line with Ritzer’s [19] understanding of consumption spaces as “cathedrals of experience,” fine dining today increasingly combines performance, emotion, and symbolism.

This research addresses that gap by focusing on Germany, a country with a distinctive regional culinary culture and an increasingly sustainability-oriented food discourse. Through a combination of media analysis and expert interviews, we aim to identify emergent themes and strategies in fine dining that reflect broader societal changes, consumer expectations, and sustainability challenges. All data used in this study were collected prior to the COVID-19 pandemic (specifically in 2018 and 2019), as the pandemic introduced significant but largely temporary effects—such as hygiene protocols, access restrictions, and short-term operational adjustments—which we deliberately excluded to avoid distorting long-term trend analysis.

Against this backdrop, there is a need for an integrated exploration of how emerging values—such as sustainability, regionalism, and narrative experience—are shaping the conceptual foundations of fine dining. This study addresses this gap by systematically mapping the interplay of thematic innovation in Germany’s high-end gastronomy through qualitative, pre-pandemic data.

## 2. Subjects and Methods 

### 2.1. Approach by Means of Qualitative Research

To explore future restaurant concepts in fine dining, a mixed-methods qualitative approach was employed, consisting of media content analysis and semi-structured expert interviews. The rationale for this approach lies in its ability to uncover both explicit and latent meanings in culinary innovation and trend development. Content analysis was performed using Mayring’s inductive category development, which allowed for both frequency analysis and thematic clustering [20]. Experts were selected based on their diverse roles within the German fine dining ecosystem—chefs, sommeliers, critics, and producers—offering a wide lens on potential futures. This approach aligns with exploratory future studies and grounded theory traditions.

For the purpose of category frequency analysis, we applied basic descriptive statistics using Microsoft Excel for Mac (Version 16.63.1), which enabled systematic counting and tabular comparison of category occurrences across all data sources.

### 2.2. Selection of Literature and Media Content

We delimited the literature coverage until the start of the COVID-19 pandemic, which inherently started new, but mostly temporary, topics like customers’ hygiene and access control, tax issues, and applications for subsidiaries. To be still the most recent, we covered the years 2018 and 2019 and referred to two leading innovative culinary specialty journals on the German market: “*Effilee*” is a quarterly magazine with a circulation of 25,000 copies. According to its own information, it deals, among other things, with “portraits of gastronomic personalities and interviews with experts from the scientific environment of the subject of culinary arts” [21]. *Port Culinaire* had a circulation of 15,000 copies at this time. “The edition Port Culinaire combines the passion for the best food and luxury food, ambitious gastronomy and exquisite and extraordinary destinations in itself,” according to the editors [22]. In addition, all episodes of the documentary series *Chef’s Table* of 2018 and 2019 were analyzed, i.e., seasons 1, 2, 3, 5, and 6. Season 4 was not analyzed, as only one specific topic (desserts) was covered here. The documentary series was produced by David Gelb and deals with international chefs and their work [23]. Furthermore, the lists “The World’s 50 Best Restaurants” of 2018 and 2019 were analyzed. This list is published by the British magazine “*Restaurant Magazine*” once a year and has a panel of 1080 culinary experts with a “structured and audited voting procedure” and describes itself as a “barometer for global gastronomic trends” [24].

### 2.3. Selection of Experts for the Personal Interviews

Representative experts from various sectors of the top gastronomy scene in Germany were selected for the interview surveys to cover a wide spectrum of relevant areas and fields of expertise. They included two top chefs, a wine sommelier, a beer sommelier, an owner of a deli mail order company, a futurologist, a food photographer and journalist, a food scout, a food producer, and an aspiring chef in top gastronomy. The experts were contacted via email and appointments were made for the interviews voluntarily. The experts are presented below with a brief description in Table 1.

### 2.4. Methodological Approach

In preparation for the expert interviews, we created interview guidelines. In the interview run-up, an analysis of specialty journals and media and extensive online research was carried out to define the framework for the expert interviews. A catalog of 14 questions was created to encourage as free a narrative style as possible on the part of the interviewees in the sense of narrative prompting questions, according to Helfferich [35]. For example, question number 2 is “How do you get to know what new trends might be?”. Furthermore, additional questions were included to keep the conversation going and also control questions to specify the topic of conversation, for example, question number 5 is “In your opinion, what are the most important trends in the next few years?”. The guide is attached to this paper as Supplementary Materials. The interview duration was not fixed. Interview duration was on average 2.5 h, with a range from 10 min to 4 h and 40 min. The interviews were then transcribed according to Kuckartz’s transcription system [36]. Thus, a verbatim transcription took place. Since space was deliberately left for as free a flow of speech as possible, passages that were clearly off-topic were omitted. Dialects and longer oral sentence constructions were partially adapted to written German for the sake of better analysis. No corrections were made with regard to grammar or word choice to retain the authentic tone and possible nuances. We employed qualitative content analysis according to Mayring for both studies. Qualitative content analysis is an evaluation method which allows analysis of data collections like interview texts, as in our case [37]. Both elements, i.e., “content” and “quality,” are of importance. In the modern understanding of content analysis, in addition to pure content, the aspects of latent content and subjective content should also be highlighted [37]. The “qualitative” part is explained in terms of a two-step process. In the first step, as in this paper, categories inductively developed from the text material are allocated to specific text passages, and in the second step, it is then necessary to check whether certain categories can be assigned to text sequences or segments more than once [37]. According to Mayring, qualitative content analysis can be summarized as follows: The object of analysis is any form of fixed communication, proceeding systematically, rule-guided, and theory-guided. The goal is to reach conclusions about specific communication aspects [20]. In this paper, therefore, the interview transcripts of the expert interviews and the content of the selected specialty journals and media were subjected to a process of “qualitatively oriented category-guided text analysis” [37]. In relation to this work, this means that the formation of categories emerged from the material under study, for example, the transcripts of the expert interviews.

In this work, the frequency analysis is of particular importance. The flow steps here are the following: The research question is the possible derivation of the restaurant concepts of the future in the field of fine dining through the analysis of the literature review and expert interviews. Subsequently, all material from the sample mentioned above was coded according to the category system, and the respective categories’ occurrence was documented in the form of a table. As an additional step of analysis, the frequency of naming of the respective category per issue, season, or expert was counted. The presentation of the results is in the form of tables with the frequencies of the names of the categories.

Thus, to address these questions, we employed a qualitative mixed-methods approach. Our study combined a structured content analysis of culinary media sources (*Port Culinaire, Effilee*, and The World’s 50 Best Restaurants) with ten semi-structured expert interviews. The analysis was based on Mayring’s [20] method of qualitative content analysis and followed an inductive logic with semi-quantitative category counts.

## 3. Results

### 3.1. Results from ”Port Culinaire” 2018–2019

After researching the gastronomy magazine “*Port Culinaire*” in 2018 and 2019, a clear trend emerged regarding which restaurant concepts we can expect to see in top gastronomy in the future (Table 2). Note that “+” indicates that a specific conceptual category was mentioned or discussed in the respective issue of *Port Culinaire* (Issues 45–52, 2018–2019). While the individual issue contents are not elaborated here, they include thematic essays, chef portraits, and trend analyses relevant to fine dining innovation. The table summarizes which categories appeared in which issues to highlight recurring patterns. Four concepts were highlighted in particular and are very closely linked. The theme-based concept, regionality, vegetarianism/veganism, and from-farm-to-table will continue to accompany us in the future: “[…] engaging with one’s own resources and recognizing that good cuisine starts on the doorstep, so to speak—with the products of the area, with traditional products of the land, with the reworking of popular tastes that have developed and endured over a long period of time” [38]. The concepts of ecology/sustainability and seasonality were also among trends that top chefs will place a lot of emphasis on: “De facto, however, it is now more in the direction of ecology, sustainability, organic and down-to-earth, a little bit in the direction of sensible cuisine in a broader sense” [38]. The future restaurant visit will not be without good and profound storytelling and deep experience because both will make the guest’s visit more memorable and trigger different emotions: “Guests definitely want to take something with them, to come out of the restaurant smarter. They want stories” [39]. Issues such as climate change and the preservation of our planet prompt us to think about the concept of new foods: “Sooner or later, insects will become part of our diet. However, not for culinary reasons, but rather out of necessity” (Issue 47, p. 135). There will also be a focus on care and recognizing the individual needs of the guest. Some top chefs will engage with the back-to-the-roots concept. For all other concepts (See Table 2), which have only been mentioned once, the question remains open as to how they will develop further.

### 3.2. Results from Effilee 2018–2019

The 2018 and 2019 editions of “*Effilee*” showed a clear trend for the future in high-end gastronomy (Table 3). The concepts of regionality and seasonality clearly stood out. Chefs feel it is a matter of course to care for our planet sustainably and to extract the maximum pleasure from the immediate environment: “Regionality and seasonality have been important keywords there for some time now—two more megatrends” [40]. “Where will the culinary journey take us in the future? Sustainability, traceability, simplicity” [41]. In addition to the concepts just mentioned, there was also a lot of talk about seasonality: “Things stay on point throughout, they are optimal at the moment they are eaten. This way of cooking is a celebration of the moment” [40]. A newcomer among the concepts was mindfulness. In our fast-paced society, this theme is guaranteed to take root. “It means paying attention to each spinach leaf or radish and preparing it in a way that develops its full potential in the process, no matter how fresh or wilted it comes into the kitchen” (Issue 44, p. 84). Trends that are closely linked to the concept of ecology/sustainability, and that we will increasingly encounter in the future, are additionally from-nose-to-tale and vegetarianism/veganism. Nostalgia and theme-based concepts will also ensure success in the future. “Tastes and flavours can connect us, with experiences from our childhood, but equally with the people we share the meal with” (Issue 50, p. 77). Among the other 16 categories mentioned once or twice each in the eight issues, the concepts of simplicity and spirituality were very exciting as they could have great potential in the future. “A highly topical food for our times and absolutely future-proof” (Issue 44, p. 86).

### 3.3. Results of ”The World’s 50 Best Restaurants 2018 and 2019

In this part of the results, “The World’s 50 Best Restaurants” and their corresponding concepts from 2018 and 2019 were analyzed (Table 4), as described in the methods section. Table 4 summarizes which conceptual categories were identified in the full descriptions of the 50 restaurants listed in The World’s 50 Best Restaurants rankings for 2018 and 2019. The inclusion of a category (“+”) reflects its presence in the restaurant’s profile, regardless of the list title or ranking position. Regionality was an obvious trend of 2018 and 2019, followed by the theme-based concept and seasonality. Chefs want to identify with their roots and traditions and present their homeland to their guests on their plates. For this, it is essential for them that their products come from the immediate surroundings, are seasonal, and represent their country. In addition to the main trends mentioned above, the concepts of from-farm-to-table and ecology/sustainability also go hand in hand. These are also topics that play a central role for the chefs. Also ripe for the future will be the multi-sensory, fusion, and show-accompanying categories. Molecular cuisine is also represented in some restaurants and could remain a transparent concept in top gastronomy. The “The 50 World’s Best Restaurants” lists also included restaurants representing the art-based and variety concepts. There were not many of them, but at least some successful representatives. Nostalgia and vegetarianism/veganism also showed an upward trend.

### 3.4. Results “Chef’s Table”

In this results section, the series “*Chef’s Table*” and its seasons 1–3, 5, and 6 were analyzed (Table 5) as described in the methods section. In the successful documentary series *Chef’s Table*, regionality was mentioned in practically all episodes. It seems that the popularity of the chefs is characterized by this category. “When you go to the source of things, you can identify with them. You understand how cooking is done and once you make those connections, you automatically orient yourself back” (Season 2, Enrique Olvera, 00:36:46–00:37:00). The concept of ecology/sustainability is something that many top chefs think about regularly: “I realised that in order for traditional Thai cuisine to continue to exist in Thailand, you have to do environmental protection” (Season 4, Bo Songvisava, 00:41:31–00:41:40). Another dominant identification of many top restaurants is the theme-based concept. This is related to the representation of its region and thus to the first concept: “Guests came from everywhere to experience Peru through its menu” (Season 3, Virgilio Martinez, 00:42:09–00:42:14). Surprisingly, nostalgia was mentioned very often. It was often emphasized how guests like to remember the past and their childhood. “In my creations, I try to call my guests back to those special moments in the past. I want them to remember their childhood through this” (Season 1, Massimo Battura, 00:10:37–00:10:46). In line with this category, the concepts of storytelling and experience were also mentioned. For many chefs it is important to represent their products through stories and information: “I don’t serve a menu, I serve a story” (Season 2, Dominique Crenn, 00:04:54–00:04:56). They strive to make every restaurant visit memorable. “Guests always have the same reaction. Facial features freeze, maybe a frown, they are indecisive for a moment and briefly irritated, then a real storm of enthusiasm follows” (Season 4, Albert Adria, 00:04:30–00:04:44). A moderate frequency was found for another three concepts that one would not immediately expect when thinking about the future of high-end gastronomy. A trend like new foods will find increased attention, simply because the world population will increase and food will become scarcer. “If everything was available at all times, I don’t think you would eat ants. If no one was ever hungry, no one would have tried worms” (Season 2, Enrique Olvera, 00:16:05–00:16:20). Furthermore, there will be an intense focus on the concept of individuality of the guest, as the needs of the guest will become even more prominent. “We always make notes here about what each guest has ordered. What they drank, what they like or what they would like to try. We keep a record of every guest who has been here” (Season 1, Niki Nakayama, 00:13:55–00:14:11). In addition, top chefs will try to direct the guests’ conscious focus on the products themselves. To do this, they will expand the concept of mindfulness more. “The dishes are simple. I keep it that way so that the idea of what this is basically about doesn’t get lost. The guests should get a feeling for the food. The thought afterwards is supposed to linger” (Season 1, Dan Barber, 00:15:38–00:15:54).

### 3.5. Results and Discussion of the Database Search in Web of Science

A database search was conducted in Web of Science using the following search terms within title, abstract, and authors’ keywords: fine dining AND future; fine dining AND concepts; fine dining AND novelties; fine dining AND development; gourmet restaurant AND future; gourmet restaurant AND concepts; gourmet restaurant AND novelties; michelin restaurant AND future; michelin restaurant AND concepts; michelin restaurant AND novelties; restaurant concepts AND future. The search did not return any matching results. This was the main reason why we used more popular media and expertise-based knowledge hubs.

### 3.6. Results from Expert Interviews

In this section of the project, expert interviews were conducted with leading figures in the field of fine dining (Table 6). The personalities were briefly introduced in the methods section. The frontrunners in the quantitative citation of categories in the expert interviews were clear: ecology/sustainability, regionality, and storytelling. A dominant theme across all expert interviews was the growing significance of sustainability as a guiding framework for future-oriented gastronomy. Several interviewees emphasized that ecological considerations are no longer optional but increasingly define purchasing decisions, menu design, and even business identity. This development is particularly evident in the preference for regional sourcing and the avoidance of products with poor ecological footprints—such as out-of-season imports or excessive animal-based components. As several interviewees emphasized, this shift toward ecological responsibility is already shaping concrete decisions: “Five years ago, I told myself: we have to buy completely differently—no more tropical fruit, no airfreighted goods, no products from greenhouses in Spain—that’s no longer acceptable,” as one expert noted (Johannes King, ll. 286–288). Another added: “We don’t serve strawberries in winter—period. If it’s not in season, it’s not on the menu. And our guests respect that” (Tobias Bätz, ll. 150–152). Another clear finding was that fine dining is shifting from a purely culinary performance toward an integrated guest experience. Interviewees repeatedly stressed the importance of storytelling and emotionally resonant spatial concepts. Rather than focusing exclusively on the plate, restaurants are becoming curated environments where architecture, service, and even soundscapes are orchestrated to evoke meaning and memory. The importance of this shift toward immersive guest experiences was captured succinctly in the words of one expert: “What we do in gastronomy is staging, dramaturgy—it’s an experience” (Johannes King, ll. 367–368). Another interviewee similarly stressed: “You don’t just come for the food—you come to be taken into another world, to feel something. The concept has to carry that” (Johannes King, ll. 290–292).

Torsten Pistol illustrates the latter, for example: “The whole hard core food project is based solely on childhood memories” (Pistol interview 28–29). He tries to use storytelling to connect with his guests’ childhood memories. According to Johannes King, storytelling will become much more conceptual and focused on one theme. For storytelling to be used successfully, this concept should be combined with another concept that has been mentioned several times, namely regionality, according to Markus Raupach, for example. This aspect can be found in numerous interviews, namely the linking of storytelling with regionality and ecology/sustainability. According to Bärbel Ring, for example, the restaurant visit should be memorable in terms of the experience.

Several interviewees highlighted that fine dining increasingly acts as a mirror and microcosm of broader socio-cultural transformations. They described how restaurants are no longer insulated luxury spaces but serve as platforms for negotiating values such as sustainability, identity, and social responsibility. This is reflected in more inclusive menu concepts, collaborations with local producers, and a growing awareness of the performative and communicative power of food. As one expert phrased it, fine dining “has become a way to tell stories about the world we live in.”

Other categories mentioned 4–5 times were experience, individuality of the guest, technologization, and vegetarianism/veganism. “Vegetables are definitely the meat of the future,” says Ralf Bos. The following categories followed with an accumulation of three mentions: Nostalgia, multi-sensory, classics, and from-nose-to-tail.

## 4. Discussion

The findings from this study reveal two distinct but interrelated thematic clusters that are shaping the future of fine dining in Germany (Table 7). This dual structure reflects earlier observations that innovation in haute cuisine often oscillates between product-centered creativity and experience-oriented transformation [3,4]. This supports earlier and recent insights that innovation in haute cuisine is embedded in institutional and symbolic contexts [3,12]. The first cluster—focusing on food itself—highlights the increasing relevance of sustainability-driven principles such as regional sourcing, ecological awareness, seasonality, and plant-based cuisine. These patterns reflect broader consumer expectations and regulatory pressures around environmental stewardship. These findings resonate with recent empirical work on sustainable fine dining experiences. Bonfanti [18] shows that sustainability-related practices are not merely seen as ethical commitments but actively contribute to the memorability and distinctiveness of high-end dining experiences. This supports our interpretation that ecological and ethical values increasingly become performative and experiential elements in the future of fine dining. In this regard, our findings support the argument that fine dining increasingly serves as a laboratory for broader societal shifts—including sustainability discourses—that extend beyond culinary boundaries [8]. This supports the idea that high-end gastronomy serves as a structured innovation environment—what Capdevila et al. [10] describe as a “creative field” embedded in broader cultural and organizational networks. As our findings further suggest, fine dining increasingly positions itself as a space of cultural leadership—echoing what Svejenova et al. [11] describe as “institutional entrepreneurship” in haute cuisine.

The second cluster, centered on the dining experience, illustrates a shift toward personalization, immersive storytelling, and emotional engagement. Notably, these concepts are not mutually exclusive: they represent two facets of a broader cultural transformation where ethical consumption and experiential authenticity converge. Our data suggest that future fine dining experiences will be defined not only by *what* is served but by *how* and, foremost, *why* it is served, making narrative, sensory design, and thematic cohesion essential elements of success. The experience-focused cluster aligns with what Ritzer [19] describes as an “enchantment of consumption,” in which dining becomes a multi-sensory, narrative-driven ritual beyond pure nourishment. This resonates with emerging cultural consumption theories emphasizing symbolic value, emotional resonance, and identity signaling. This interplay of ethical substance and experiential form aligns with previous research on the symbolic and emotional dimensions of fine dining, where dining is framed as a form of identity signaling and cultural meaning making [5,6]. Thus, this study contributes to both hospitality research and broader debates in consumer culture theory by highlighting fine dining as a critical site of aesthetic, ethical, and cultural negotiation.

The two thematic clusters—sustainability-oriented food practices and experience-driven dining—differ in focus and audience appeal but offer distinct forms of potential. The sustainability cluster addresses widely relevant concerns such as ecological responsibility, plant-based cuisine, and regional sourcing, which are increasingly reflected in broader consumer expectations. The experience-oriented cluster, by contrast, emphasizes emotional engagement, storytelling, and immersive settings, contributing to fine dining’s role as a cultural and aesthetic practice. Both approaches show potential in terms of audience development and economic value, depending on the strategic goals and identity of the restaurant. Rather than being mutually exclusive, they may also intersect in innovative hybrid formats.

How could the future of restaurants in fine dining be further elucidated? In the study presented here, the trends described above emerge as hypotheses for the future, both in the specialty journals/media and in the expert interviews. Of course, this work also has limitations, such as the focus on Germany, the qualitative methodological approach, or the comparatively small number of experts. Other possibilities for testing the hypotheses generated here would be, for example, a standardized survey of visitors to gourmet restaurants on possible future concepts because, after all, the future also depends heavily on the wishes and ideas of customers. Also conceivable would be a real simulation or video-based presentation of the individual categories worked out here with test persons and a subsequent survey with quantitative evaluation of the results. Other think tanks would also be possible, where food manufacturers, chefs, restaurateurs, restaurant guests, nutrition experts, futurologists, and business leaders could discuss new impulses.

This research sheds light on how fine dining in Germany is likely to evolve in response to changing cultural values, ecological imperatives, and consumer aspirations. Our findings underline the need for restaurants to go beyond culinary excellence by embedding sustainability and narrative depth into their core offerings. While limited to a qualitative scope and Germany-specific context, this study offers a conceptual foundation for future cross-cultural, comparative, and quantitative research on food innovation and experiential consumption. Such contextual explorations reflect recent calls in the literature to examine haute cuisine within specific national gastronomic systems rather than treating it as a universally homogeneous category [2,9]. As fine dining continues to operate at the vanguard of gastronomic culture, it holds a mirror to larger societal shifts—a mirror that scholars and practitioners alike should not overlook. Future research could explore how these trends manifest in different cultural contexts or are perceived by diners themselves.

Building on the identified thematic clusters, we propose that future fine dining businesses align their strategies with three core measures: First, integrating sustainability visibly into sourcing and storytelling can meet ethical expectations without sacrificing exclusivity. Second, cultivating individual guest experiences through data-informed personalization and immersive concepts can enhance emotional engagement. Third, long-term success will require structural adaptability—chefs and operators must be open to continuous innovation, interdisciplinary collaboration, and proactive engagement with societal shifts. These measures may offer a pathway for fine dining to remain both culturally relevant and economically sustainable in the years ahead. These strategic directions echo findings from earlier innovation-focused studies in haute cuisine, which emphasize the need for constant adaptation, creative synthesis, and human-driven transformation in high-end gastronomy [3,4].

Future research should build on these findings through cross-cultural comparisons, quantitative validation, and guest-centered studies to further refine our understanding of evolving gastronomic expectations.

## Figures and Tables

**Table 1:** Presentation of the experts.

Expert	Brief Description of the Person
Torsten Pistol	Torsten Pistol is the head of Pistole Hardcore-Food GmbH (food production), a company he founded, which is intensively involved with regional concepts, disseminates this philosophy, and organizes events to match [25].
Markus Raupach	Markus Raupach is a historian, journalist, publisher, author, marketing expert, and sommelier for beer, fine spirits, and cheese. He is co-founder of the German Beer Academy in Bamberg and the president of the German Beer Consumer Union (GBCU) [26].
Willy Faber	Willy Faber is known as the publisher and managing director of the magazine “*Gastronomie Report*.” He is also the initiator of the “Restaurant of the Future” competition and can be described as a futurologist [27].
Tobias Bätz	Tobis Bätz is a German 2-Michelin-star chef (plus a Green Michelin star) and head chef at the Romantisches Posthotel in Wirsberg. This restaurant (now known as AURA) was founded by 2-Michelin-star chef Alexander Herrmann. With the dual leadership of the above two people, the restaurant received a second Michelin star in 2019 [28].
Bärbel Ring	Bärbel Ring has been a sommelière at Söl’ring Hof on Sylt since 2009, and in October 2015 she was named best sommelière of the year [29].
Ralf Bos	Ralf Bos is the founder of Bos Food GmbH, which was established in 1990. With a product portfolio of about 12,000 gourmet articles, Ralf Bos and his team supply gourmet addresses all over Germany [30].
Jörg Osswald	Jörg (“Joshi”) Osswald is a food scout in the team of Alexander Herrmann and Tobias Bätz in the restaurant of the Romantisches Posthotel in Wirsberg. He does this full-time [31]. He is also founder of Papa Mame GbR.
Johannes King	Johannes King was head chef (accredited with 2 Michelin stars) and hotelier at Söl’ring Hof on Sylt until 2018. His focus was on regional and seasonal products, much of which he was able to take from his own garden and the island of Sylt. Since 2013, he has been running his own delicacies trade shop in Keitum [32].
Benedikt Kraus	Benedikt Kraus is a trained chef and visionary gastronomist. He has gained various experiences in various internships. He is currently working on a new restaurant concept and made “Upper Franconia’s best burger” in 2018 [33].
Thomas Ruhl	Thomas Ruhl was the editor of one of the most influential professional magazines in top gastronomy, namely “*Port Culinaire*,” which he founded in 2003. Furthermore, he is well-known as an international food photographer, and he is also the author of many professional articles and illustrated books on fine dining and future concepts of cooking [34].

**Table 2:** Frequency of designations of categories in *Port Culinaire* from 2018–2019.

	Theme-Based Concept	Regionality	From-Farm-to-Table	Vegetarianism/Veganism	Storytelling	Experience	Ecology/Sustainability	Seasonality	New Food	Individuality of the Guest/Individual Needs of the Guest	Back-to-the-Roots	Zero Waste	Simplicity	Technologization	Nostalgia	Multi-sensory	Art-Based	From-Nose-to-Tail	Meat Substitute	Fermentation
Issue 45	+		+	+			+		+	+	+		+	+						+
Issue 46	+	+	+	+	+	+	+	+		+						+	+			
Issue 47	+	+	+		+	+	+	+	+			+		+					+	
Issue 48	+	+																		
Issue 49				+				+												
Issue 50	+	+			+	+			+	+					+					
Issue 51	+	+	+	+	+	+	+	+	+		+							+		
Issue 52	+	+	+	+			+				+	+	+		+					
Quantity	7	6	5	5	4	4	4	4	4	3	3	2	2	2	2	1	1	1	1	1

**Table 3:** Frequency of category designations in *Effilee* from 2018–2019.

	Regionality	Ecology/Sustainability	Seasonality	Mindfulness	From-Nose-to-Tail	Nostalgia	Vegetarianism/Veganism	Theme-Based Concept	Experience	Fermentation	Variety	Storytelling	Back-to-the-Roots	Zero Waste	New Food	Art-Based	Craftsmanship	Simplicity	Spirituality	Technologization	Fusion	From-Farm-to-Table	Meat Substitute	Individuality of the Guest/ Individual Needs of the Guest
Issue 44	+	+		+	+			+	+	+				+			+	+	+					+
Issue 45	+	+	+	+	+	+		+								+					+			
Issue 46	+		+								+		+											
Issue 47	+	+		+			+			+														
Issue 48	+	+	+									+	+											
Issue 49	+	+																						
Issue 50	+	+	+	+		+	+		+		+									+		+		
Issue 51	+	+			+	+	+	+				+			+								+	
Quantity	8	7	4	4	3	3	3	3	2	2	2	2	2	1	1	1	1	1	1	1	1	1	1	1

**Table 4:** Frequency of category designations in “The World’s 50 Best Restaurants” lists of 2018 and 2019.

	Regionality	Theme-Based Concept	Seasonality	From-Farm-to-Table	Ecology/Sustainability	Multi-sensory	Fusion	Molecular Cuisine	Show-Accompanied Program	Art-Based	Variety	Nostalgia	Vegetarianism/Veganism	Back-to-the-Roots	Zero Waste	From-Nose-to-Tail	History	Storytelling	Fermentation	New Food	Technologization
List 2018	23	14	4	4	5	4	3	3	4	2	2	1	2	1	0	1	1	1	0	0	1
List 2019	27	11	14	8	4	4	5	5	2	4	4	3	2	2	3	2	1	1	2	1	0
Quantity	50	25	18	12	9	8	8	8	6	6	6	4	4	3	3	3	2	2	2	1	1

**Table 5:** Frequency of named categories in the show *Chef’s Table* seasons 1, 2, 3, 5, and 6.

	Regionality	Theme-Based Concept	Nostalgia	Storytelling	Ecology/Sustainability	Experience	Mindfulness	Individuality of the Guest	New Food	Craftsmanship	Show-Accompanied Program	Variety	Fermentation	Art-Based	From-Nose-to-Tail	Back-to-the-Roots	Seasonality	Spirituality	Molecular	From-Farm-to-Table	Quality	Vegetarianism/Veganism	Multi-sensory
M. Battura (S1)	+		+											+									
D. Barber (S1)	+				+		+													+			
F. Mallmann (S1)						+										+							
N. Nakayama (S1)	+					+		+									+						
B. Shewry (S1)	+		+	+																			
M. Nilsson (S1)	+											+	+										
G. Achatz (S2)			+			+		+			+								+				
A. Attala (S2)	+	+		+	+	+			+														
D. Crenn (S2)		+	+	+										+									
E. Olvera (S2)	+	+							+														
A. Ros (S2)	+	+																					
J. Kwan (S3)	+	+			+		+						+					+				+	
V. Mukhin (S3)	+	+	+																				
N. Silvertone (S3)	+									+											+		
I. Orkin (S3)																							
T. Raue (S3)	+	+																					
V. Martinez (S3)	+	+		+	+				+														
C. Martinez (S5)										+					+								
M.Dagdeviruses (S5)	+	+																					
B. Songvisava (S5)	+	+	+		+																		
A. Adria (S5)						+					+												+
M. Bailey (S6)	+	+	+	+																			
D. Cecchini (S6)															+								
A. Khan (S6)		+		+				+				+											
S. Brock (S6)	+	+	+	+	+		+																
Quantity	17	13	8	7	6	5	3	3	3	2	2	2	2	2	2	1	1	1	1	1	1	1	1

**Table 6:** Frequency of the categories in the expert interviews, ordered by decreasing number of mentions.

	Ecology/Sustainability	Regionality	Storytelling	Experience	Technologization	Individuality of the Guest/ Individual Needs of the Guest	Vegetarianism/Veganism	Nostalgia	Multi-sensory	Classics	From-Nose-to-Tail	Theme-Based Concept	Back-to-the-Roots	From-Farm-to-Table	Health	Show-Accompanied Program	Streetfood Cuisine	Seasonality	New Foods	Zero Waste	Fermentation	From-Sweet-to-Sour	Mindfulness
Torsten Pistol	+	+	+			+		+															
Markus Raupach	+	+	+	+		+	+								+		+						+
Willy Faber	+	+	+	+	+				+			+	+			+							
Tobias Bätz	+		+					+		+													
Bärbel Ring	+	+	+	+	+		+			+	+												
Ralf Bos	+	+	+	+			+				+												
Jörg Osswald	+	+			+					+	+												
Johannes King	+	+		+	+							+			+			+					
Thomas Ruhl	+	+	+		+	+	+		+				+	+		+			+	+			
Benedikt Kraus	+	+	+			+		+	+					+							+	+	
Quantity	10	9	8	5	5	4	4	3	3	3	3	2	2	2	2	2	1	1	1	1	1	1	1

**Table 7:** Two main topic blocks.

Thematic Block 1: Concerns Food Itself	Thematic Block 2: Concerns Eating Out
Regionality	Storytelling
Ecology/Sustainability	Nostalgia
Seasonality	Theme-based concepts
From-farm-to-table	Experience
Vegetarianism/Veganism	Mindfulness/Guest individuality

## Data Availability

The original contributions presented in the study are included in the article, further inquiries can be directed to the corresponding author.

## Supplementary Materials

The following supporting information can be downloaded at: https://www.mdpi.com/article/10.3390/foods14132294/s1, Table S1: Systematic catalogue of questions for the expert interviews.
