# Supplementary material for: Envisioning the Future of Fine Dining: Insights from a Multi-Methods Study in Germany
Source: Foods. 2025 Jun 28;14(13):2294. doi: 10.3390/foods14132294 (PMC12249383; doi:10.3390/foods14132294)
Supplement: Supplementary file 1 [file foods-14-02294-s001.zip › foods-3694431-supplementary.pdf]

**Table S1:** Systematic catalogue of questions for the expert interviews.

| **Questions for the expert interviews (14 questions in total)** |
|---|
| 1) Are there any constants that people and customers simply always like or at least have liked for a very long time? ("What is always good?") |
| 2) How do you find out what new trends might be?<br>- How do you track them down?<br>- Which tools, media or people help you to do this?<br>- What is a trend that is worth following and which is not? |
| 3) How do you manage to set a new trend?<br>- Which new restaurant or indulgence concepts are characterised by their own signature? |
| 4) What is your philosophy for success, life and job? |
| 5) What do you think will be the most important trends in the coming years? |
| 6) Why do you think these will be successful topics? |
| 7) What are you guided by? The competition, your own instincts or do you follow trends? |
| 8) What role does psychology play in your area of specialisation? |
| 9) What role does storytelling play? |
| 10) What inspires you in your daily work and what inspires you for the future?<br>- How do chefs get inspired? Where do they get their inspiration from? |
| 11) What challenges will there be in the future? |
| 12) What are your worries and fears? |
| 13) How do you see the future of top gastronomy? |
| 14) What is your ultimate motivation for doing your job? |

Note: Questions 2,3 and 10 have follow-up questions.
